# PHL14/352: The Impact of Electronic Health Information and Computer-mediated Communication for the Coping Abilities of Cancer Patients

**DOI:** 10.2196/jmir.1.suppl1.e94

**Published:** 1999-09-19

**Authors:** A Scheiber, M Gruendel

**Affiliations:** ^1^University for Applied Sciences, MagdeburgGermany

**Keywords:** Health Promotion, Internet, Health Communication, Electronic Networks, Self-help Groups

## Abstract

**Introduction:**

World wide-based Information services are providing an increasing variety in information concerning practically every aspect of prevention, health promotion and treatment for patients, their families and care providers. For the case of cancer, a well-established information and communication infrastructure for an English speaking audience has been developed over the past years.

**Methods:**

Our work has focused on an extensive literature review, identifying publications on the impact of the use of electronic information and communication platforms of the Internet by cancer patients, their families and care providers.

**Results:**

Patients mainly log on to major services to receive background information about their particular disease or general introductions about the nature of cancer. The question of how much patients' ability to access to more information about their disease really effects their quality of life is still open. A randomised controlled trial showed that patients appreciated receiving more written information on cancer, although this did not increase their knowledge about cancer. Besides following their wishes or needs to inform themselves via the WWW, patients use more often email and newsgroup bulletin boards for communication. These bulletin boards also function as screening agencies for cancer news. Questions concerning the treatment of cancer and its effects on the family are reported as the most helpful topics on those lists. The three major dimensions of communication goals in online discussion groups can be identified as follows: a) exchange of information, b) social support and c) personal empowerment. These findings are similar to the communication structures identified for face-to-face groups. However, there are indications for some differences between traditional and virtual self-help (VS): more people are involved in VS; VS is open not only for patients but for their families, care providers as well as for involved professionals; VS is accessible around the clock and from every place.

**Discussion:**

At this point of time, we can only estimate the importance of the Internet for health communication and health promotion. However, it appears that the Internet is a large information resource as well as a potential support network for patients and their families. In virtual groups, the distance-intimacy-relation seems to be easier to control. Furthermore, membership is independent from regional circumstances and patients with very rare kinds of cancer are able to find peers. The latter leads to the question if VS is particularly attractive to difficult-to-reach patient groups and if it will enhance their access to important resources such as social support. The process of coping with cancer itself seems to be very well supported by such new non-traditional electronic support networks, as long as they fulfil the functions of a community. Some basic problems, concerning the quality of information and the reliability of medical advice available through email, are not yet solved.

